# Automatic relevance detection in the absence of a functional amygdala

**DOI:** 10.1016/j.neuropsychologia.2011.02.032

**Published:** 2011-04

**Authors:** Dominik R. Bach, Deborah Talmi, René Hurlemann, Alexandra Patin, Raymond J. Dolan

**Affiliations:** a Wellcome Trust Centre for Neuroimaging, University College London, United Kingdom; b School of Psychological Sciences, University of Manchester, United Kingdom; c Department of Psychiatry, University of Bonn, Germany

**Keywords:** Amygdala, Relevance detection, Emotion

## Abstract

The idea that the amygdala is crucially involved in automatically prioritising relevant events rests on evidence from a single lesion study where a patient with bilateral temporal lobe lesions, acquired in adulthood, was impaired in recall facilitation during the attentional blink. Here, in a comparable task, we show that two individuals with selective bilateral amygdala lesions retain facilitated recall of aversive words during the attentional blink. Recall facilitation was statistically significant for both patients and akin to that seen in young students and age- and education-matched controls. This challenges the amygdala's role as a crucial hub in prioritising attention and at a minimum implies that this role can be compensated for when lesions are acquired early in life. Previous findings might be explained by the described fact that lesions were acquired later in life and encompassed areas beyond the amygdala, including visual pathways. We propose that in the absence of a functioning amygdala, prioritised visual processing may rely on alternative structures such as pulvinar and cortical visual areas.

## Research highlights

► We investigate two patients with complete and specific amygdala lesions due to Urbach-Wiethe syndrome. ► In an emotional attentional blink task, they show no impairment of automatic relevance detection. ► This opposes earlier findings of a patient with surgical temporal lobe lesion in adulthood. ► Cortical and thalamic visual areas might support prioritised resource allocation in the absence of a functioning amygdala.

## 1 Introduction

The amygdala is believed to facilitate an evaluation of motivationally relevant sensory events (Bach, Schachinger et al., 2008; Sander, Grafman, & Zalla, 2003; Zald, 2003). In particular, fMRI data show that low-level appraisal activates the amygdala (e.g. Bach, Grandjean et al., 2008; Critchley et al., 2000; Hariri, Bookheimer, & Mazziotta, 2000; Hariri, Mattay, Tessitore, Fera, & Weinberger, 2003). Such observations have fostered the idea that the amygdala serves as a computational hub that prioritises the processing of relevant events by quick, low-level, allocation of processing resources towards these events (Pessoa, 2008). A paradigmatic example of the latter is provided by recall facilitation for arousing stimuli seen in the attentional blink (AB) paradigm. Here, participants are tasked to detect two target stimuli in a rapid serial visual presentation (RSVP) stream of distractor objects. When a second target (T2) occurs within a relatively short temporal interval after the first (T1), its recall is impaired (Raymond, Shapiro, & Arnell, 1992). The AB effect per se is thought to reflect competition between processing of the T1 item with distractor items at a relatively late processing stage, where all stimuli have preserved processing at an early sensory stage (Chun & Potter, 1995). This preserved early processing provides the basis for an attenuation in the AB seen when T2 is an emotionally arousing word (Anderson, 2005; Martino, Strange, & Dolan, 2008; Keil & Ihssen, 2004), reflecting a facilitation in processing of motivationally relevant items.

In a previous report, patient SP with bilateral temporal lobe lesion failed to show facilitation of emotional T2 items, suggesting amygdala integrity is necessary for an automatically prioritised processing of emotional events (Anderson & Phelps, 2001). Contrary to expectations based on this proposal it has recently been shown that patient SM, with bilateral amygdala damage, was not impaired in some tasks requiring automated attention allocation to fearful faces, but was impaired in explicit recognition of fear expressions (Tsuchiya, Moradi, Felsen, Yamazaki, & Adolphs, 2009). To reconcile these findings we note that patient SP (Anderson & Phelps, 2001) acquired her right amygdala lesion through neurosurgery at the age of 48 years, involving areas beyond the amygdala, at which time her left amygdala lesion was also discovered. On the other hand, patient SM (Tsuchiya et al., 2009) suffers from Urbach-Wiethe syndrome. This is a congenital disorder of the brain and skin that leads to a relatively specific amygdala calcification, the latter encroaching gradually during childhood and adolescence (Newton, Rosenberg, Lampert, & O’Brien, 1971).

In these previous reports on SM and SP, the tasks assessed different aspects of prioritised processing and therefore do not afford a direct comparison. Hence, in the present study we sought to directly assess facilitated recall in the attentional blink (i.e. the task used in SP) in the 34 year old monocygote twins BG and AM, diagnosed with Urbach-Wiethe syndrome at the age of 12 after BG had an epileptic seizure (Hurlemann et al., 2007; Talmi, Hurlemann, Patin, & Dolan, 2010). We hypothesised that these patients would show significant recall facilitation, in contrast to SP.

## 2 Methods

### 2.1 Design and participants

The experiment followed a followed a 2 (T2 valence: neutral/aversive) × 3 (T1–T2 lag: 1, 2, 3) factorial design. Two 34 year old German-speaking female twins with congenital lipoid proteinosis (Urbach-Wiethe disease), previously characterized by Hurlemann et al. (2007) and Talmi et al. (2010), took part in the study. While BG suffered a single epileptic grand-mal seizure aged 12, AM has never suffered an epileptic seizure.

Clinical cranial computer tomography showed bilateral calcification lesions that symmetrically span the whole amygdala region (see Fig. S1 in supplemental material). Both patients are of average intelligence (L-P-S Leistungsprüfsystem) (Horn, 1983) and exhibit intact performance in a neuropsychological test battery that includes verbal learning and memory (Rey Auditory Verbal Learning test) (Helmstedter, Lendt, & Lux, 1981), executive function measured with the Trail Making Test (Reitan, 1955), Wisconsin Card Sorting Test (Kongs, Thompson, Iversion, & Heaton, 2000), Stroop test (Bäumler, 1985), semantic fluency (Aschenbrenner, Tucha, & Lange, 2000), and show neither depression nor anxiety (Hamilton, 1959, 1960). The patients exhibit limited neuropsychological impairments in phonemic fluency (Aschenbrenner et al., 2000) and in d2-test, a sustained visual cancellation task tapping short-term concentration (Brickenkamp, 1995). AM is impaired in figural learning and memory, as shown in the Complex Figure Test (Osterrieth, 1944) and the DCS (Weidlich & Lamberti, 2001).

We compared these patients to a control group of female university students (*N* = 42, age 23.3 ± 4.6 years) and an age- and education-matched control group of females from the general population (*N* = 23, age 35.7 ± 3.8). Duration of formal education of this group was 13.0 ± 0.1 years (13 years for both AM and BG). In contrast to patients, this latter group showed average performance on the L-P-S and d2 test, but results from these tests did not co-vary with performance in the AB paradigm (*p* < .20). The study was approved by the research ethics committee at the University of Bonn.

### 2.2 Stimuli and experimental paradigm

We used a 10 Hz rapid serial visual presentation [RSVP] paradigm (70 ms presentation/30 ms pause) of German nouns where distractor items appeared in black and target items in white on a grey background. After a fixation cross (950 ms) and 5, 10, or 15 black distractor words (0.66° vertical angle) on grey background, a white T1 was presented, followed by either 0, 1, or 2 distractor words, the white T2, and 5 more distractor words. Participants were tasked to freely recall the target stimuli within 3 s after the trial ended with their performance recorded for off-line comparison to actual stimulus presentations. The experiment was broken up into 6 blocks where stimulus order was randomised. Each of 72 T1 and 72 T2 stimuli was used – in different order and parings – in three non-consecutive blocks. Ninety-nine neutral words served as distractor items and were recycled randomly.

Neutral (T1, T2 and distractor) and negative-arousing (T2) German nouns were drawn from a large validated database (Hager & Hasselhorn, 1994) and in-house material. The two words in each T1–T2 pair had the same number of syllables and different first letters. First letter frequency was the same in neutral and aversive T2 items, and both subsets had the same mean word length (*p* > .50). Frequency of the words in general usage was determined in April 2005 using a publicly available database (http://wortschatz.uni-leipzig.de) that is based on a large number of newspaper articles (Quasthoff, 1998) and was not different between neutral and aversive words (*p* > .50). A list and detailed description of the word material can be found in Bach (2009).

### 2.3 Statistical analysis

For the control group, T2 recall accuracy after successful T1 recall was calculated for each participant and each cell of the design, and subjected to 2 (valence) × 3 (lag)—repeated measures ANOVA on the group level. Within-subjects inference for the two patients was drawn using a random permutation test on trials where T1 was recalled correctly. Under the null hypothesis that valence is not associated with recall, we randomly permuted (10,000 repetitions) valence labels within each lag and counted the number of recalls for both valence labels across all three lags in order to empirically determine the null distribution. For each patient, a *p*-value was derived as the ratio of permutations where null recall advantage was higher than the actual recall advantage. Under the null hypothesis that neither of the two patients shows recall facilitation, the combined *p*-value is obtained by multiplying the two individual *p*-values and states the error probability for the alternative hypothesis that at least one, or both, patients benefit from recall facilitation. Similarly, the effect of lag was assessed by permuting the lag labels within valence labels, and the interaction by permuting both valence and lag labels.

The deviation of the patients’ scores from the control samples was conducted using Bayesian Monte Carlo methods (Crawford & Garthwaite, 2007) as implemented in the software *SingleBayes* (http://www.abdn.ac.uk/∼psy086/dept/Programs/SingleBayes.exe). This provides a point estimate of the percentage of the control population that would obtain a lower score (i.e., a point estimate of the abnormality of the score) and a 95% credible interval for this quantity.

## 3 Results

**Fig. 1:**
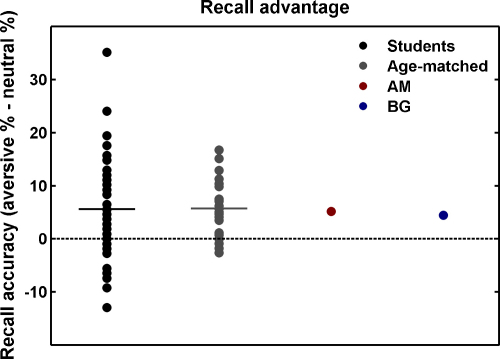
Recall facilitation (% correctly recalled aversive T2 − % correctly recalled neutral T2) for both control groups and patients AM and BG. Patients’ recall facilitation is almost identical to the control group mean and is significantly different from zero (see text for details).

In both control groups, T2 recall was dependent on T1–T2 lag (students: *F*2, 82 = 42.8; *p* < .001; age-matched controls: *F*2, 44 = 17.6; *p* < .001) with an inverted U-shape, as reported previously (Raymond et al., 1992). More importantly, both control groups showed a main effect of valence (students: *F*1, 41 = 15.9; *p* < .001; age-matched controls: *F*1, 22 = 25.5; *p* < .001), and no valence × lag interaction (see Fig. 1, see also Fig. S2, supplemental material). The latter finding was expected because we tested the early T2 lags only, during which the AB facilitation for emotional words is most robust (Anderson, 2005; Anderson & Phelps, 2001; De Martino et al., 2008; Keil & Ihssen, 2004).

Strikingly, both patients showed almost precisely the same effect of lag and valence as the mean of the control groups. For both patients together, the valence effect was significant at *p* = .02 (permutation test). This shows that at least one, or both, of the two patients benefitted in recall facilitation for aversive items, strongly arguing against a suggestion that this is necessarily compromised in patients with amygdala lesions. Similar to healthy people, the effect of lag was significant at *p* < .0001, but there was a valence × lag interaction (*p* < .0001).

Next, to show that the size of this effect was not different from the healthy population, we estimated the population ratio that would have lower recall facilitation than our two patients. For the different combinations of patient and control group, an estimated 40.1–52.9% of the normal population would have lower recognition facilitation than our patients, and the lower bound of 95% confidence intervals around these estimated ratios was between 25.6% and 36.3%. This means that with 95% confidence, at least one quarter of the population would have lower recall facilitation than the patients. This speaks against any impairment in our patients and is strikingly different from SP's performance that was below the level of any of the control individuals under study (Anderson & Phelps, 2001). Also, neither the effect of lag nor the interaction was different from the respective effects in the control group. The fact that the interaction was significant in the patients but not in the control group is explained by the use of more sensitive non-parametric testing in patients.

## 4 Discussion

In two patients with complete and selective amygdala damage, we report significant recall facilitation for aversive words in the attentional blink. This is in pronounced contrast to a previous case study that failed to find such recall facilitation in patient SP with extended medial temporal lobe damage (Anderson & Phelps, 2001) which has led to the idea that amygdala integrity is necessary for automatic relevance detection. Our finding is more in keeping with a recent report that patient SM with selective amygdala damage is not impaired in automatic processing of fearful faces which also constitute relevant events (Tsuchiya et al., 2009).

The idea of the amygdala as a relevance detector (Sander et al., 2003) is based mainly on a plethora of fMRI experiments showing increased BOLD responses to relevant as opposed to non-relevant stimuli, where relevance includes, but is not limited to, emotional (positive or negative) arousal and motivational value (see Zald, 2003 for a comprehensive review). A key idea here is that relevance detection is part of a low-level (i.e. automatic and pre-conscious) appraisal system (Scherer, Schorr, & Johnstone, 2001), and consequently is has been shown that BOLD responses in the amygdala are typically stronger when relevant stimuli are processed incidentally (also termed implicitly) as opposed to when individuals give explicit assessment of their relevance (e.g. emotional category) (Bach, Grandjean et al., 2008; Critchley et al., 2000; Hariri et al., 2000, 2003). Because of its anatomical connections (McDonald, 1998), the amygdala has been proposed to act as a computational hub, integrating and relaying information from many different brain regions (Pessoa, 2008) where one of several emergent functions is to prioritise the processing of relevant events for example by deploying attentional resources.

Although Pessoa (2008) in principle opposes the one-region-one-function view and emphasises one-to-many and many-to-one mappings, the study by Anderson and Phelps (2001) seems to suggest that complete amygdala lesions lead to catastrophic impairment in automatically prioritising the processing of relevant events, in keeping with the conceptualisation of the amygdala as a computational hub. In this framework it is surprising then that Tsuchiya et al. (2009) reported unimpaired (implicit) processing of fearful face expression in subject SM with amygdala damage, where explicit detection was impaired. Similarly, patients with unilateral amygdala damage are not impaired in a visual search task that also required implicit relevance detection of emotional targets (Piech et al., 2010). Here, we extend these observations by showing, in a paradigm similar to the one used by Anderson and Phelps, that our patients AB and BG have unimpaired recall facilitation in the attentional blink.

How can this discrepancy be resolved? The key difference between Anderson and Phelps’ and our study is lesion type, particularly its extent and its temporal onset. Patient SP's right-hemisphere amygdala lesion was the result of surgery, at the age of 48, for intractable epilepsy due to reactive gliosis that also caused a left-hemisphere amygdala lesion (Anderson & Phelps, 2001; Phelps et al., 1998). This surgical lesion in the right hemisphere involves regions beyond the amygdala encompassing the hippocampus, parahippocampal cortex, parts of the anterior, middle and inferior temporal gyri (Phelps et al., 1998) and thus important components of the ventral visual stream (Mishkin and Ungerleider, 1982). Also, the aetiology of SP's reactive gliosis appears unknown though symptoms related to this occurred as early as 3–4 years. Thus, there is a range of uncertainty as to when the amygdala lesion occurred, spanning from the earliest years of life right up to the time of surgery.

Hence, two explanations for the discrepancy between AM, BG, and SM on the one hand, and SP on the other, seem possible. One is that amygdala integrity is necessary for prioritised processing of relevant stimuli in adulthood, but that when amygdala lesions occur early in life, as in Urbach-Wiethe syndrome, adaptive mechanisms compensate for this deficit. A diametrically opposite hypothesis has been put forward based on the early observation that patients with amygdala damage due to adult herpetic encephalitis are not impaired in explicitly (i.e. non-automatically) recognising facial emotion (Hamann et al., 1996), whereas individuals with Urbach-Wiethe syndrome are. However, this latter account is not supported by a later investigation which reported impairments in emotion detection after adult herpetic encephalitis (Broks et al., 1998). Also, this finding relates to explicit processes rather than automatic relevance detection and therefore does not directly contradict our account. Another, perhaps more likely, possibility is that resource allocation to relevant events relies on medial temporal lobe structures, for example parts of the ventral visual stream within inferior temporal gyrus (or fibre bundles). These structures are lesioned in SP's right hemisphere but not in classic Urbach-Wiethe patients. It remains the case that patients with unilateral right-hemispheric temporal lesions in the study by Anderson and Phelps (2001) did not show an impairment, and patients with unilateral left temporal lesions were impaired, leading the authors to argue that the left hemisphere is of particular importance. As SP's left hemisphere shows no macroscopic damage beyond the amygdala, the amygdala was seen to be the relevant structure. However, our finding of no impairment in two patients with lesions clearly restricted to the amygdala, suggest that a network of extra-amygdala structures, might be responsible for the deficit seen in SP.

Both the above explanations require that structures other than the amygdala support automatic relevance detection and allocation of attention in our patients. One candidate is a network that includes the pulvinar and related cortical visual regions. The pulvinar is anatomically interlinked with higher visual areas and with the amygdala. These structures have been ascribed a central role in relevance detection (Pessoa and Adolphs, 2010). The ventral visual stream, which we have noted is compromised in SP, could be a crucial structure in this network. As a caveat, we realised automated relevance detection in an attentional blink paradigm with words to afford direct comparison with SP's data. Word meaning is a highly evolved ability, and our conclusions would be supported by a replication in experimental paradigms using simpler visual stimuli, for example faces with and without emotional expression.

To summarise, we find unimpaired early processing of motivationally relevant events in two individuals with complete and selective bilateral amygdala damage acquired during adolescence. In the light of previous findings, our results point to a critical role of structures in the visual system that support prioritising of resources either under normal circumstances or as compensatory mechanism when an amygdala lesion occurs early in life.
