# Supplementary material for: Automatic relevance detection in the absence of a functional amygdala
Source: Neuropsychologia. 2011 Apr;49(5):1302–5. doi: 10.1016/j.neuropsychologia.2011.02.032 (PMC3083511; doi:10.1016/j.neuropsychologia.2011.02.032)
Supplement: Supplementary file 1 [file mmc1.doc]

**Figure S1:** CCT scans of patients AM and BG as of November 2008, showing the extent of calcification in the medial temporal lobe.


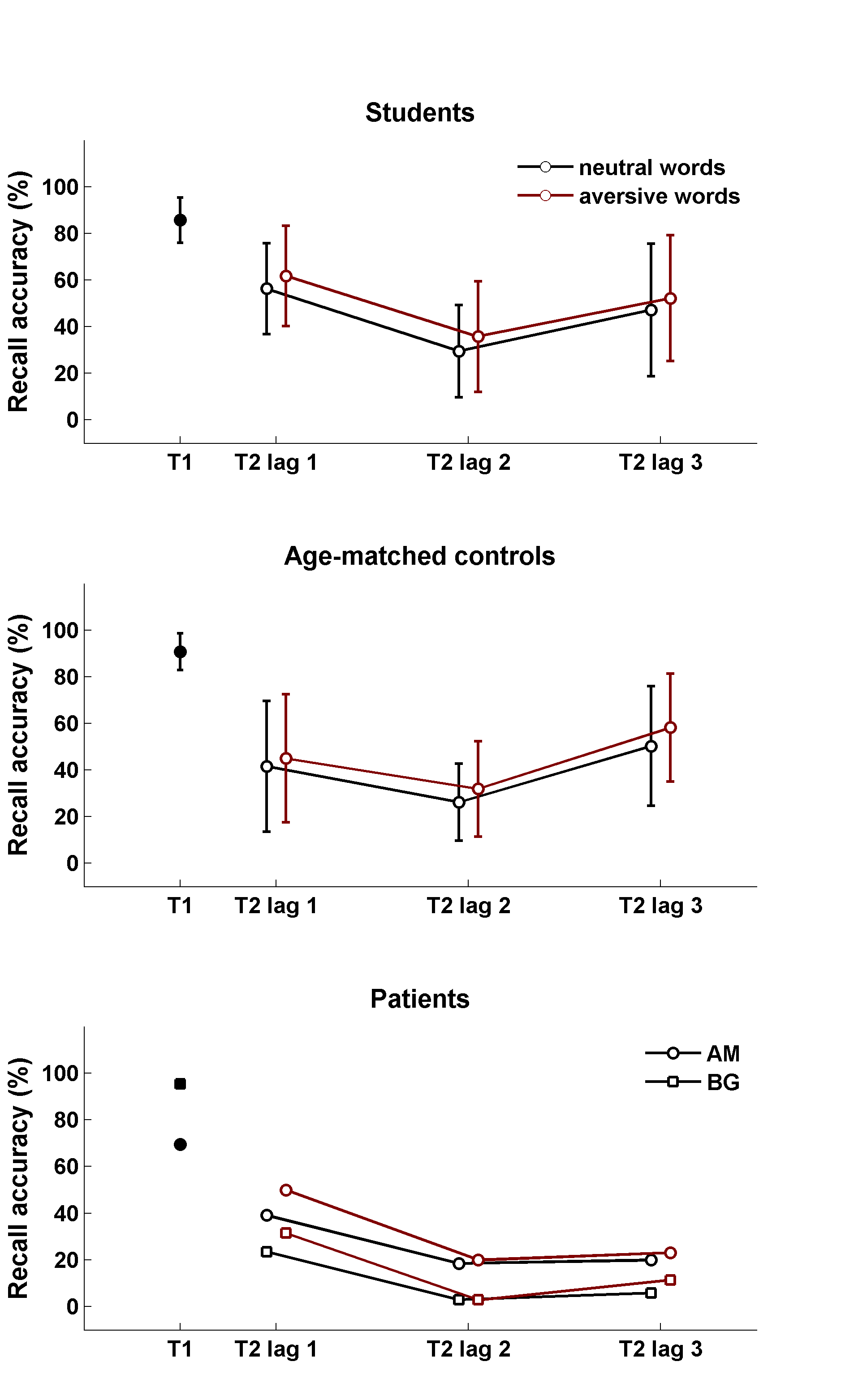


**Figure S2:** Recall accuracy in % (mean ± standard error) for T1; and for T2 after successful recall of T1, according to T1/T2 lag and T2 valence.
